# Enhancement of brackish water desalination using hybrid membrane distillation and reverse osmosis systems

**DOI:** 10.1371/journal.pone.0205012

**Published:** 2018-10-09

**Authors:** Emad Ali, Jamel Orfi, Abdullah Najib, Jehad Saleh

**Affiliations:** 1 Chemical Engineering Department, King Saud University, Riyadh, Saudi Arabia; 2 Mechanical Engineering department, King Saud University, Riyadh, Saudi Arabia; Aix-Marseille Universite, FRANCE

## Abstract

Desalination of geothermal brackish water by membrane distillation (MD) provides a low recovery rate, but integrating MD with reverse osmosis (RO) can maximize the production rate. In this study, different design configurations of a hybrid system involving brine recycling and cascading are studied via simulations, and the performance improvement due to the process integration is substantiated via the increased recovery rate and reduced specific energy consumption. Brine recycling is also found to improve the recovery rate considerably to 40% at an energy cost of 0.9 $/m^3^. However, this achievement is only valid when the final brine is recycled to the RO feed: when the final brine is recycled to the MD feed, the overall performance degrades because the recycled brine cools the feed and causes a serious reduction in the driving force and the consequent production rate. Configuring the hybrid system in multiple stages connected in series increases the recovery rate to 90% and reduces the specific energy consumption to 0.9 MJ/kg. Although the specific energy cost increases dramatically because external inter-stage heating is implemented, using a free energy source (such as a geothermal or waste-energy source) for inter-stage heating could provide the optimum configuration.

## Introduction

Geothermal energy is derived from hot water or steam drawn from sub-soil and is mainly used for generating electricity, producing heat, and cooling. Lund et al. [1] reviewed direct global applications of geothermal energy worldwide and determined that it was used for bathing and swimming, heating spaces and districts, and acted as a ground source heat pump. Specific data and information on the use of geothermal energy in 82 countries up to year 2015 were gathered, presented, and discussed. The report showed that the amount of thermal power installed globally for direct utilization at the end of 2014 was approximately 71,000 MW, and the electrical capacity of the European Union reached 993.6 MW in 2015 (with 915.5 MW in Italy) [2]. However, geothermal energy use is limited in Saudi Arabia, where there is an installed capacity of only 40 MW for bathing and swimming and 4 MW for animal farming, providing a total direct use application of 152.89 TJ/year [1].

In a recent paper, Gude [3] noted that geothermal energy sources are clean, sustainable, and act as both heat sources and energy storage systems. He discussed the current status and future prospects of geothermal desalination, and also stressed the merits and potential use of geothermal energy sources to drive thermal desalination processes, as the energy is available in large quantities (which is required by thermal desalination). Gude [3] also analyzed case studies from various countries (including Saudi Arabia, Costa Rica, and Australia) to determine the progress of technological developments made in this field.

Several studies relating to Saudi Arabia have reported relatively low enthalpy sources at temperatures lower than 100 °C [3–5]. In their study of potential geothermal energy in the kingdom, Demirbas et al. [4] compiled the characteristics of main hot spring locations in the country and discussed the potential utilization of such sources, and AlHarbi [5] conducted a survey on available geothermal energy resources in the country and classified them based on exergy using a specific exergy index (SEI). The SEI values of all identified geothermal wells were found to be very low, which means they are classified as very low-grade energy sources. Their potential uses are therefore limited to low-enthalpy applications, including heating and low-temperature desalination methods, such as LT-MED, humidification and dehumidification (HDH), and membrane distillation (MD). These low-grade geothermal energy sources are thus good candidates for driving conventional thermal desalination processes, and in this respect, low-temperature multiple effect distillation (LT-MED) using geothermal energy has been proposed in several studies. In addition, Davies and Orfi [6] proposed a framework study showing the technical feasibility of self-powered geothermal desalination of groundwater sources at temperatures lower than 100°C. Additionally, desalination processes (including MD) driven by renewable energy sources (such as solar or geothermal sources) has become an attractive concept, as reflected in the increasing number of studies conducted on such integrations [7,8].

In their review of main studies focusing on MD powered by solar energy, Qtaishat and Banat [9] reported that although it has been proven that the combination of solar energy and MD is technically feasible, the cost of water production is relatively high compared to the use of commercial photovoltaic RO. Guillen et al. [10] presented results obtained from two pre-commercial MD modules driven by solar systems under the same weather and operating conditions and discussed data on energy consumption, efficiency, and production rates. The authors noted that although the multistage concept for MD can reduce energy consumption, the production of fresh water is still low. In addition, Manna et al. [11] used cross-flow flat-plate modules to conduct experimental work on the removal of arsenic from contaminated groundwater used for drinking water by employing solar-powered MD; results showed that almost 100% of the arsenic was removed from the water. This confirms one of the major advantages of the MD process: its ability to treat various types of feed waters, even those with high salt concentrations.

However, one of the major limitations of MD is its low recovery ratio, and an increasing number of studies focused on solving this limitation have been published in the last several years. Such studies have proposed associated methods and configurations, assessed their respective effectiveness, developed new types of materials and membranes, and conducted appropriate experiments and tests. For example, Summers et al. [12] presented a fundamental study on the energy efficiency of single stage MD under different configurations of brine regeneration and energy recovery. Their results showed that air gap membrane distillation (AGMD) and direct contact membrane distillation (DCMD) have the potential to provide a high gain output ratio (GOR) if properly optimized. Summers et al. [13] also developed theoretical frameworks using various main MD configuration models, which enabled them to conduct a comparative study on the performance of these configurations. The results for single stage MD showed that, in particular, vacuum membrane distillation (VMD) is very limited, as its GOR is always lower than one. From a different perspective, Winter et al. [14] conducted an experimental investigation on flux enhancements using feed water deaeration on spiral wound MD modules. Their results showed that deaerated water can be used to remove air from the volume pores of the membranes, thereby highlighting one of the beneficial effects of such a method.

Several concepts and methods have been used to enhance MD performance, particularly with respect to increasing the recovery ratio and improving product fluxes, such as brine recycling methods, the use of energy recovery devices, and the use of hybrid desalination processes. In addition, the implementation of a multi-stage (or multi-effect) concept, which is commonly used in conventional multiple-effect distillation and multi-stage flash technologies, has received much interest and attention [15,16], as it can increase the recovery ratio and also reduce the specific energy consumption of the desalination process.

Integrating MD with other processes can improve the overall performance of the entire combined system. Macedonio and Drioli [17] designed and studied the performance of a reverse osmosis system followed by a membrane distillation unit; their results are encouraging as they present the possibility of overcoming the limitations of each single unit. Criscuoli and Drioli [18] conducted an energy and exergy analysis of an integrated system coupling RO, MD, and nanofiltration (NF) modules, in which the MD unit operated on the RO brine while the NF unit was used for RO feed pretreatment, and concluded that such an integrated system represented an attractive alternative to RO and to conventional thermal desalination processes. In addition, Mericq et al. [19] proposed an integrated VMD—RO unit (in which VMD was used as a complimentary process to RO) to further concentrate the RO discharge brines and thus increase the overall recovery of the plant. Furthermore, El-Zanati and El-Khatib [20] proposed a hybrid system consisting of NF and RO followed by VMD, where the overall recovery for seawater was increased from 30–35% when using RO to 76.2% when using the hybrid system. Pangarkar et al. [21] reviewed the coupling of RO and MD processes for desalination of groundwater and presented several advantages of using such an integrated system with groundwater in India. Zhang et al. [22] investigated the performance of a membrane distillation crystallization unit operated on brines from a seawater RO unit and obtained a water recovery ratio of 90%. Swaminathan et al. [23] proposed a theoretical analysis of a hybrid mechanical vapor compressor (MVC) and MD to reduce specific energy consumption, and Osman et al. [24] analyzed the performance of hybrid multi-stage flash distillation (MSF)–RO desalination plants for large applications.

To provide further results on integrated systems, this current paper presents a theoretical analysis of an integrated MD—RO driven partially by geothermal energy. Several integration scenarios are proposed, and results are analyzed in terms of the recovery ratio, production rate, product quality, and energy consumption.

## Modeling and simulation

In the authors’ previous study [25,26], extensive experiments were conducted on a single pilot-scale MD to analyze the process performance and develop a rigorous model; this previously validated model of the MD unit will be used here. However, previous experimental and theoretical studies performed on a single MD by the authors [25,26] revealed that the performance of this desalination system was low and needed improvement. For example, it was found that the recovery ratio and production rates are too low: the recovery ratio does not exceed a maximum value of 5%. Similar conclusions are supported by other studies in the literature. This present work proposes and assesses methods that can be used to enhance the desalination performance; for example, by integrating MD with RO and brine recycling. The feed water is considered to be brackish water with fixed properties, and the presented results therefore relate to brackish water that has the same baseline feed conditions: a hot flow rate of *Q*_*ho*_ = 300 L/h and hot water feed temperature and salinity of *T*_*ho*_ = 70°C and *C*_*so*_ = 1.414 kg/m^3^, respectively, (the latter two conditions are representative of certain wells in the Riyadh region).

**Fig 1:**
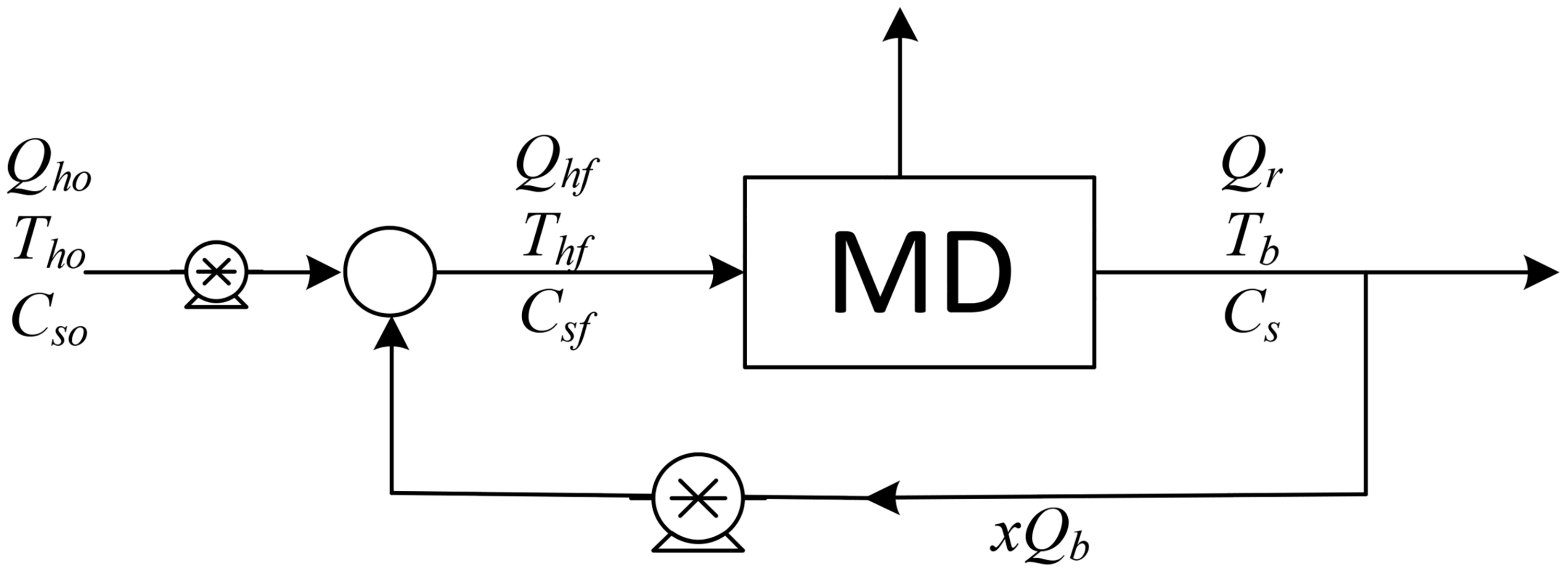
Design structure option 1.

**Fig 2:**
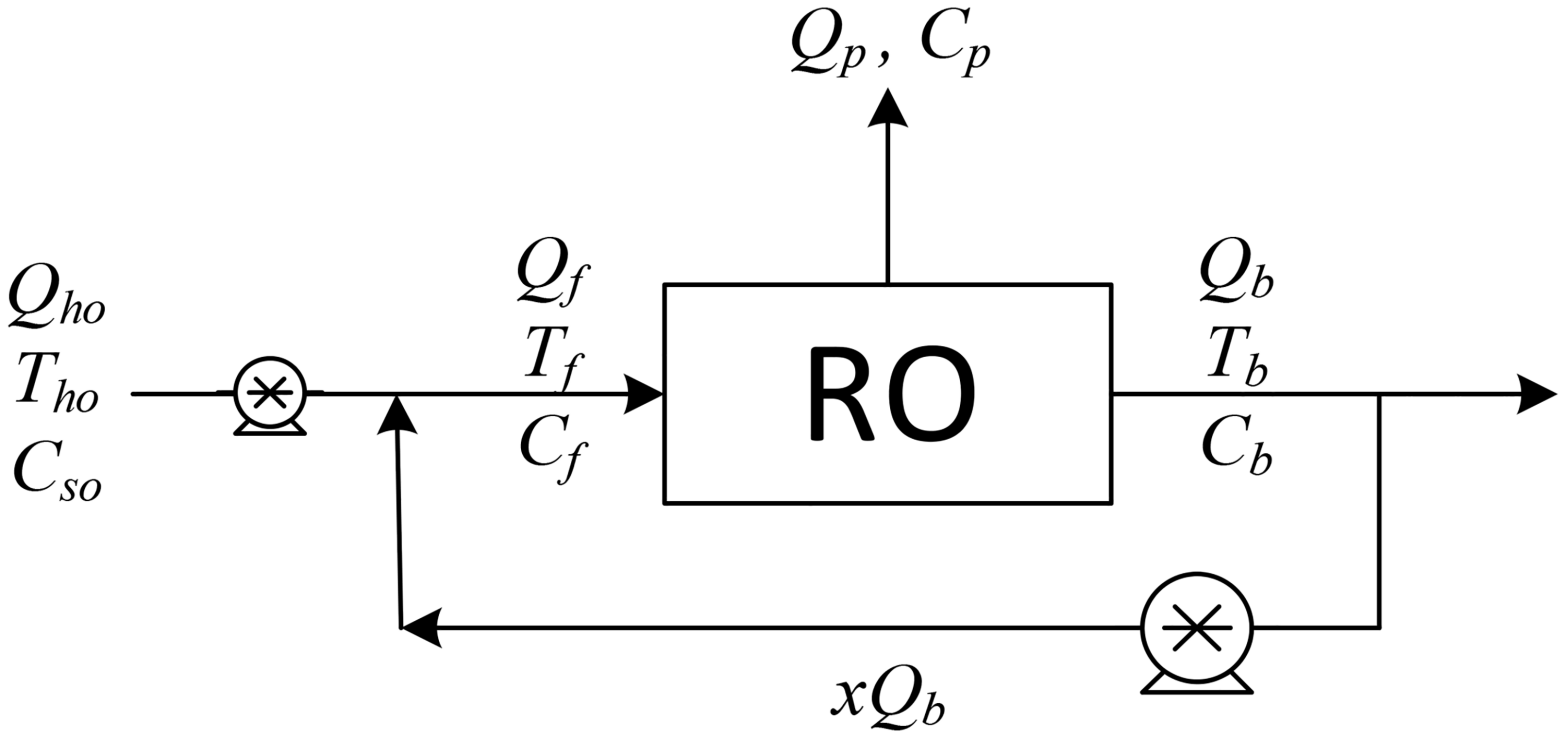
Design structure option 2.

**Fig 3:**
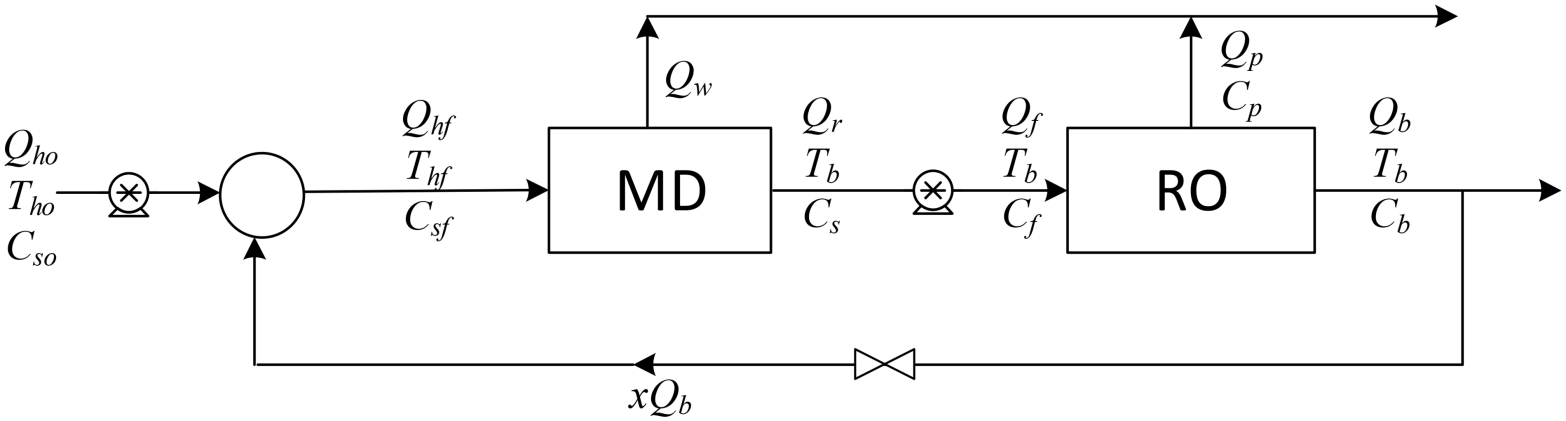
Design structure option 3.

**Fig 4:**
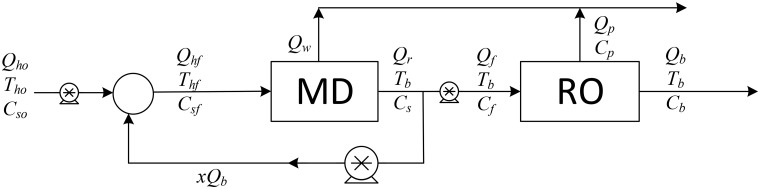
Design structure option 4.

**Fig 5:**
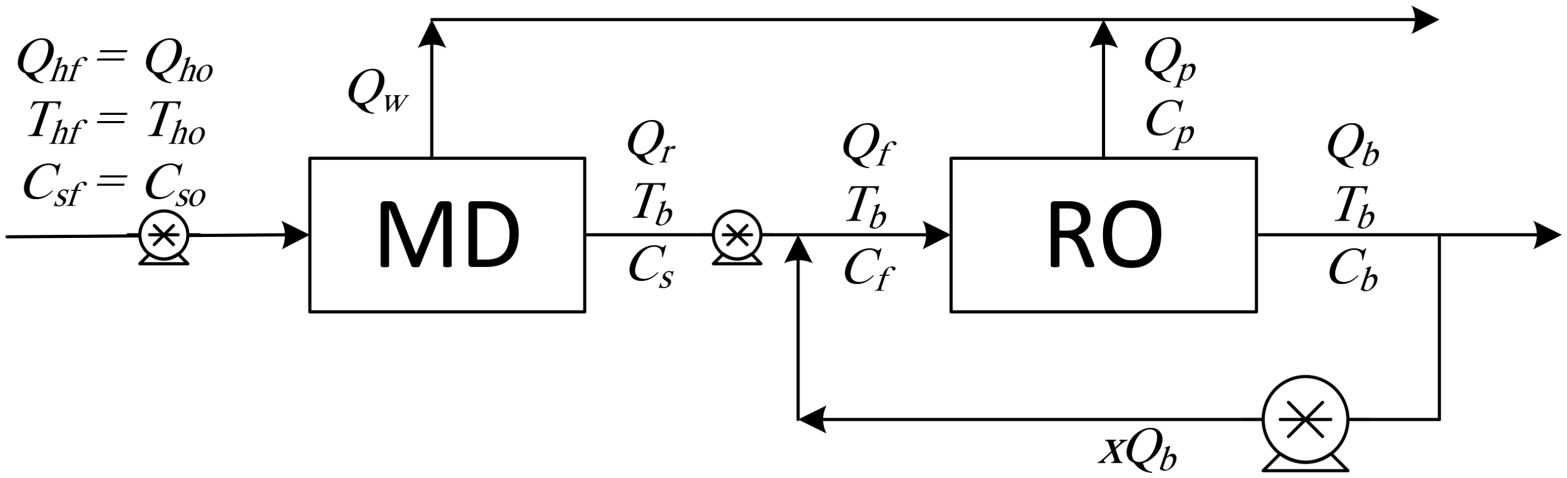
Design structure option 5.

**Fig 6:**
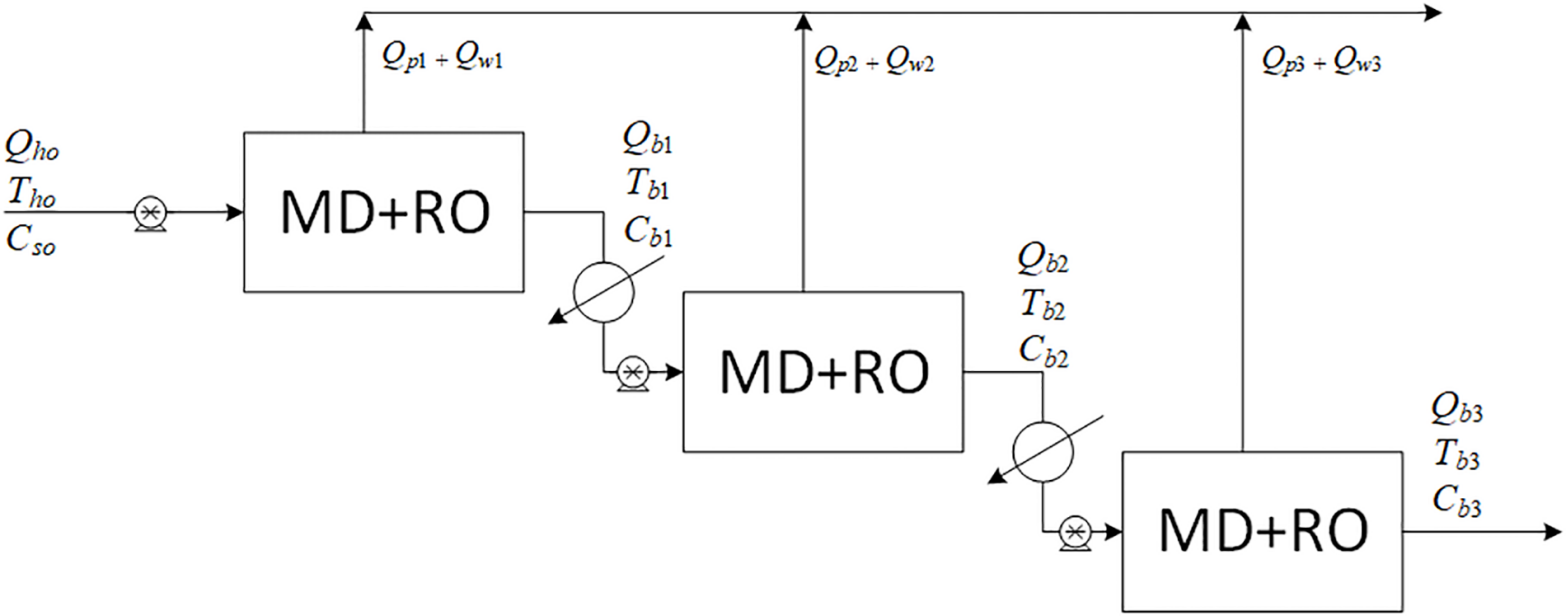
Design structure option 6.

As previously mentioned, the authors found that a single MD unit provides a low recovery ratio, and to improve the overall desalination process, we examined six different desalination configurations, which are shown in Figs 1–6.

The aim of this study is to determine the optimal design configuration that provides the best performance, and this is assessed using selected key performance indicators (KPIs). Two scenarios for feed flow rates are considered: one is to fix the fresh feed flow rate at a baseline value while allowing the feed flow rate to vary with recycling; and in the second scenario, the feed is fixed at the baseline value while the fresh feed is allowed to decrease as recycling increases. Note that the fresh feed is interchangeably called “makeup” throughout the manuscript. Table 1 summarizes the main options considered in this study.

**Table 1:** Summary of studied MD/RO with brine recycle options.

Option number	Description	Diagram	Figure
1	Single MD with brine recycle (BR)	SMDBR	1
2	Single RO with BR	SROBR	2
3	MD/RO in series with BR on both units	MDROBR1	3
4	MD/RO in series with BR on MD unit	MDROBR2	4
5	MD/RO in series with BR on RO unit	MDROBR3	5
6	MD/RO in stages	MDROSTG	6

Option 1 suggests desalinating the brackish water using a single MD unit with brine recycling to increase the production rate. Option 2 considers desalinating the brackish water using a single RO unit after cooling the salty water to room temperature, and brine recycling is also used to increase the recovery rate. Options 3 to 5 combine the benefits of MD and RO to enhance the production rate, and the options differ only in the location of the recycle stream. It is of note that although is advisable to place the MD after the RO (because the former is deployed for high salinity solutions), in this study, the MD unit is always placed in front of the RO unit, as this enables the energy associated with the geothermal water to be utilized. In addition, feeding high temperature saline water directly into the RO processes is not recommended. Option 6 is a multi-stage hybrid MD/RO system that connects the stages in series; inter-stage external heating is employed in this configuration to maintain the feed temperature of each stage at 70 °C.

In all configurations, an additional cold-water stream is used whenever MD is involved. However, it is not shown in the diagrams, both because it is independent of other streams and omitting it enables the use of simple diagrams. It is always assumed that cold water is available to cool the MD unit, and it is also assumed that no additional energy is needed to cool it. Pumps are used to maintain the required pressure in each unit: the pumps between the MD and the RO units are employed to increase the pressure to a level required by the RO system. Recycle streams are equipped with pumps to compensate for the pressure drop, although this does not occur with Option 3, where a throttling valve is used because the pressure of the brine exiting the RO is higher than the operating pressure of the MD. The KPIs chosen for an individual RO or MD unit are given in Eqs. (S.25–S.28) and (S.45–S.48), respectively. For Options 1 and 2 where recycling is enforced, the performance ratio and energy cost include the pumping energy used for recirculation. In addition, the recovery ratio is based on fresh feed. However, for the hybrid systems of options 3–5, the KPIs are modified as follows,

$$Rc={(Q}_{w}+Q_{p})/Q_{ho}$$ (1)

$$SR=1-C_{p}/C_{so}$$ (2)

$$Pfr=\frac{{\left(E_{p}\right)}_{MD}+{\left(E_{p}\right)}_{RO}+{\left(E_{p}\right)}_{rec}+H_{in}}{{(Q}_{w}+Q_{p})\rho }$$ (3)

$$E_{cost}=\frac{[{\left(E_{p}\right)}_{MD}+{\left(E_{p}\right)}_{RO}+{\left(E_{p}\right)}_{rec}]\times e_{c}}{Q_{w}+Q_{p}}$$ (4)

Option 6 consists of staged processes connected in series, where each stage comprises a single MD followed by a single RO without recycling. Although Fig 6 shows three stages in series for illustration purposes, the number of stages can be much larger than three. For option 6, when several stages with preheating are used, the KPIs are written as follows,

$$Rc=\frac{\sum_{i=1}^{n}{\left(Q_{w}+Q_{p}\right)}_{i}}{Q_{ho}},$$ (5)

$$SR=1-\frac{\sum_{i=1}^{n}{C_{p}}_{i}}{C_{so}},$$ (6)

$$Pfr=\frac{\sum_{i=1}^{n}\left[{\left(E_{p}\right)}_{MD,i}+{\left(E_{p}\right)}_{RO,i}+H_{in,i}\right]}{\sum_{i=1}^{n}{\left(Q_{w}+Q_{p}\right)}_{i}\rho },$$ (7)

$$E_{cost}=\frac{\sum_{i=1}^{n}\left[{\left(E_{p}\right)}_{MD,i}+{\left(E_{p}\right)}_{RO,i}+{\left(E_{p}\right)}_{rec,i}\right]\times e_{c}+\sum_{i=2}^{n}H_{in,i}\times e_{h}}{{\sum_{i=1}^{n}\left(Q_{w}+Q_{p}\right)}_{i}}.$$ (8)

For Option 6, it should be noted that the cost of heating duties for the second and subsequent stages is included in the overall energy cost because external heating sources are utilized. In addition, the heating duty of the first stage is excluded because it is provided naturally by the geothermal source. In the equations above, the cost of electricity is taken as *e*_*c*_ = 0.06 $/kwh and that of heating is taken as *e*_*h*_ = 13.3 $/Gj, which is the cost of saturated steam at 5 bar and 160 °C [27].

**Fig 7:**
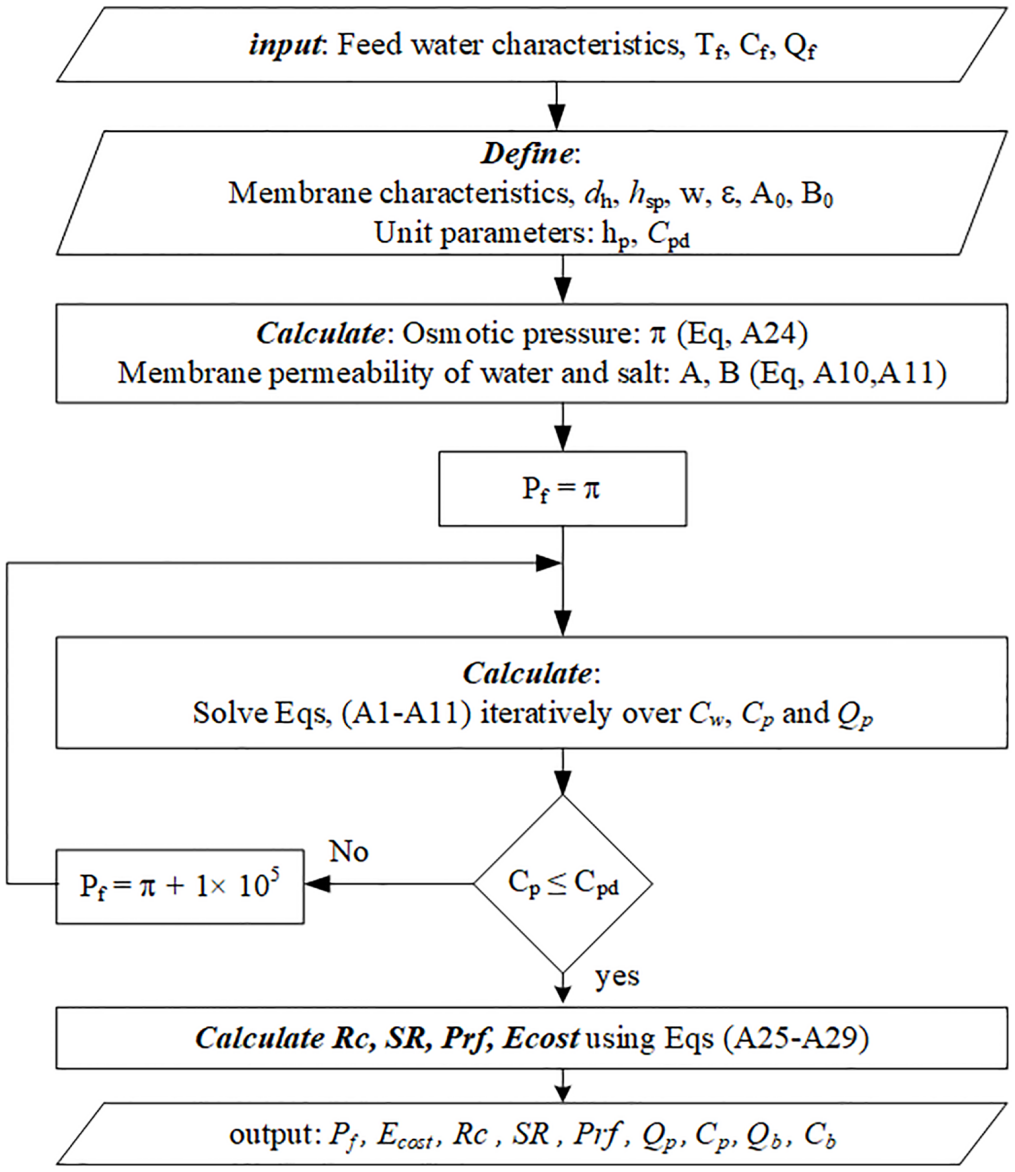
Organigram 1, RO model solution algorithm.

**Fig 8:**
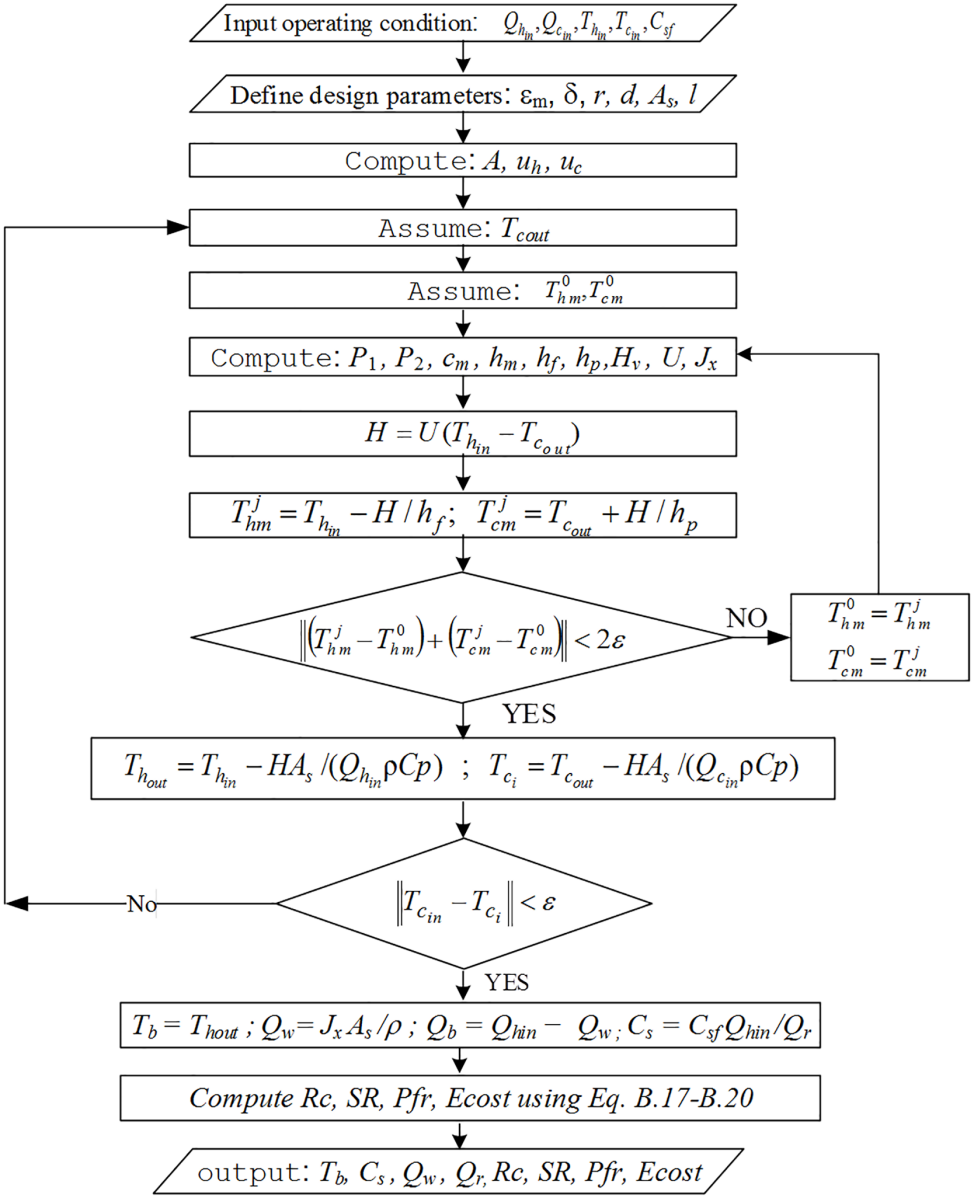
Organigram 2, MD model solution algorithm.

**Fig 9:**
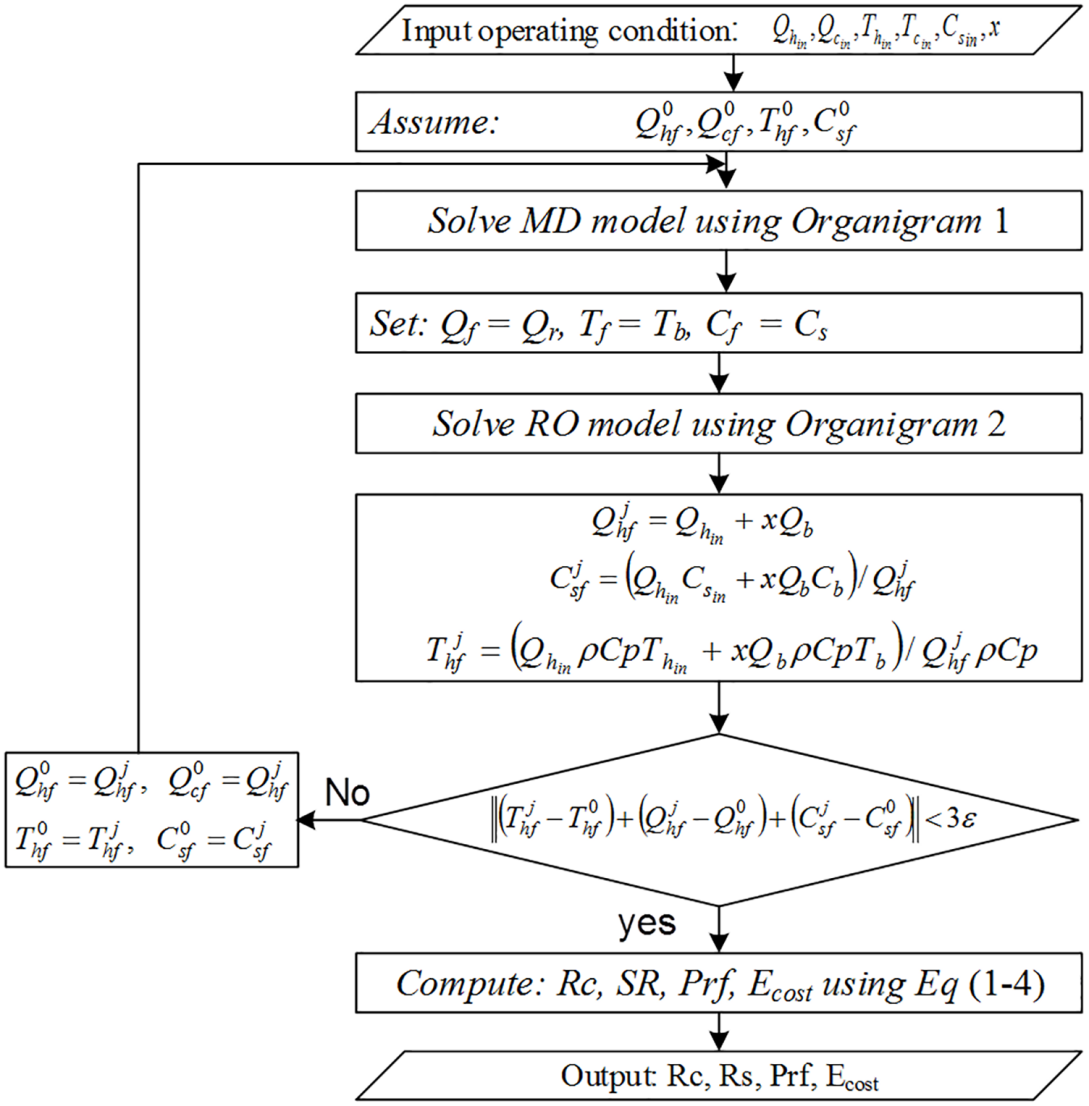
Organigram 3, algorithm for design structure Option 3.

Simulations conducted using these different design structures are shown using the organigrams in Figs 7–9. Organigram 1 shows the core algorithm for a single RO unit without recycling. The underlying equations describing the water separation inside a typical RO membrane are solved iteratively for a given feed pressure, and the resultant permeate salinity is checked against the desired water quality. However, if the constraint is not satisfied, the pressure is increased monotonically until the required specification is met. Similarly, organigram 2 illustrates the core solution routine for a single MD unit without recycling. The mass and heat transfer equations are solved simultaneously and iteratively; when solutions converge, the exit temperature and flow rate are computed using simple overall mass and energy balances. Organigram 3 demonstrates the simulation of design structure Option 3, where recycling affects the feed conditions. Specifically, the feed flow rate, temperature, and salinity change during recycling, and therefore, the algorithm iterates over these parameters until they converge to a steady value for a given recycle ratio. The given algorithm is suitable for a fixed makeup flow at a baseline value and with a varying feed flow rate. For a case when the makeup is allowed to vary with recycling, the feed is fixed at the baseline value and excluded from the iteration loop. Furthermore, the makeup is calculated as the difference between the feed and recycle streams. In the same fashion, a similar algorithm for design structures 1, 2, 4, and 5 can be developed. However, for simplicity and because of space limitations, these algorithms are not presented.

## Results and discussion

**Fig 10:**
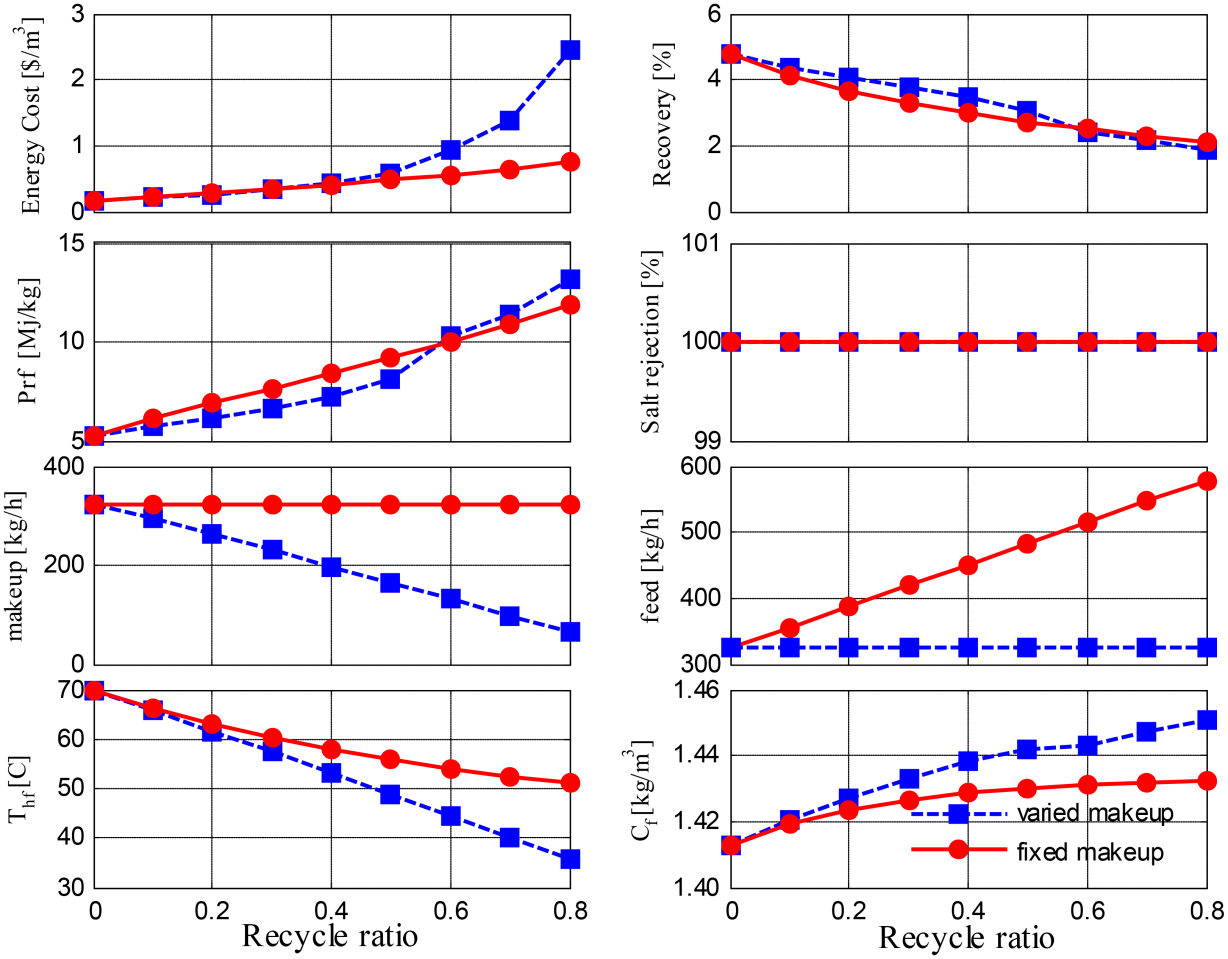
Performance of Option 1 versus recycle ratio.

Fig 10 depicts the simulation results for Option 1 at a range of recycling ratios for both fixed and varying makeups, and the results show that the recycling ratio has an obvious effect on the salinity and temperature of the MD feed. Typically, for a large-size MD, as used in this study, the brine leaves the MD at a low temperature; therefore, recycling the brine reduces the feed temperature substantially, which degrades the driving force at the membrane interface and hence results in reduced water production. Consequently, the recovery ratio diminishes as the recycle ratio increases, which then decreases the performance ratio and increases the specific energy demand. The situation deteriorates further when a varying fresh feed is adopted. With a decrease in the makeup flow rate, less energy is supplied to the system; this leads to a sharp drop in the feed temperature, which then causes a considerable degradation of mass production. Although water production is not shown in the figure, its effect is reflected in the specific energy and performance ratio, which grow exponentially at higher recycling rates. Although energy for pumping is constant for the fixed feed flow and corresponds to a varying makeup, the associated water production decreases rapidly, which leads to a sharp increase in specific energy cost. Interestingly, variations in the recovery ratio with changes in the recycling ratio are almost the same for both varying and fixed makeups, and this occurs because water production and makeup ratios remain almost the same due to their relative change rates. It is of note that pure water is obtained, which means that there is 100% salt rejection. Nevertheless, the performance obtained using Option 1 is not promising.

**Fig 11:**
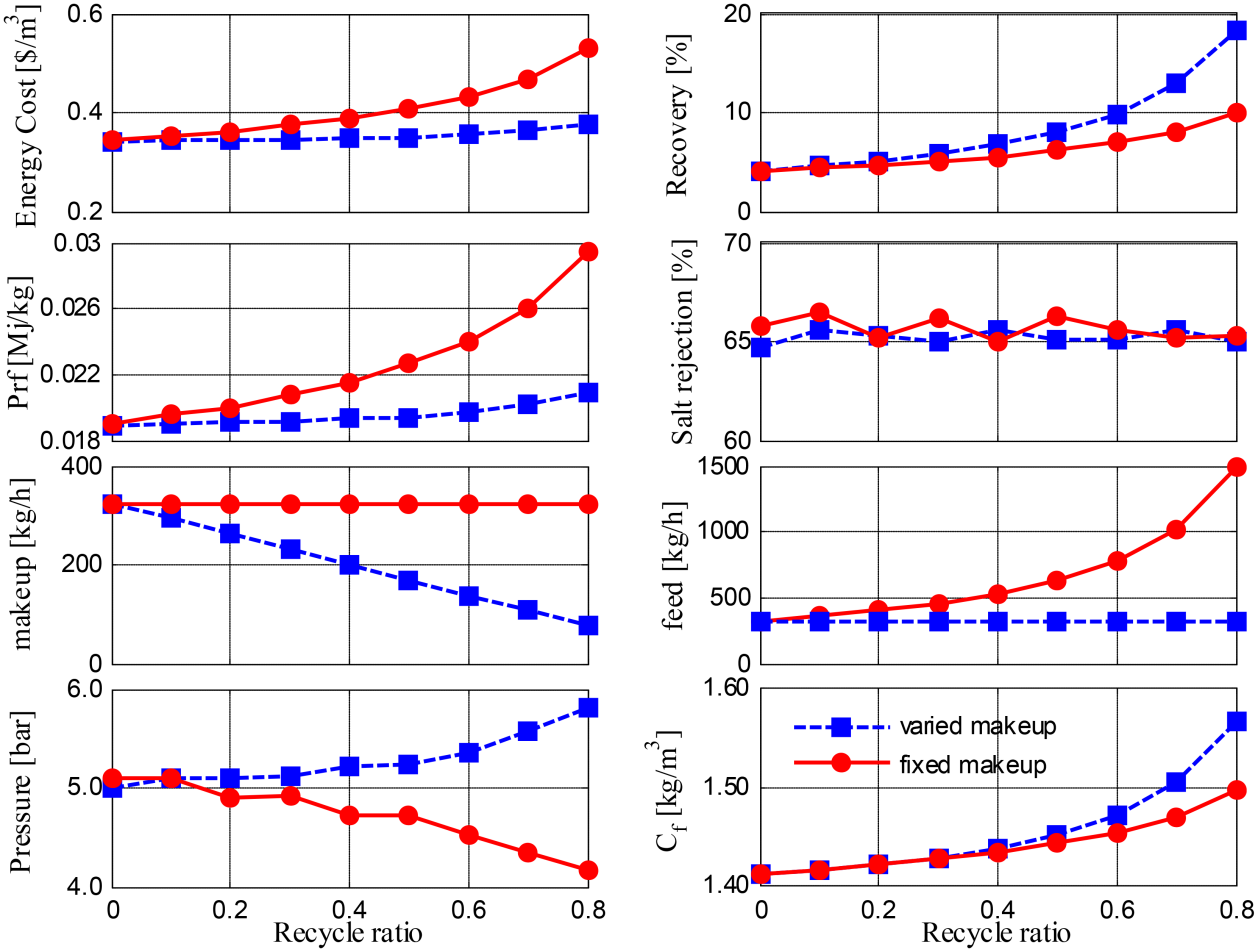
Performance of Option 2 versus recycling ratio.

The use of RO with brine recycling is the most commonly used practice for desalinating geothermal water after it is cooled. Its use was thus tested, and simulation results corresponding to Option 2 are depicted in Fig 11. As more brine is fed back, there is no doubt that Option 2 outperforms Option 1 in terms of the increase in the recovery rate. This configuration also requires less specific energy and has a lower cost. Although the pumping energy requirement for RO is higher than the pumping energy requirement for MD, because RO operates at higher operating pressure, the associated water production of RO is greater, which leads to lower specific energy and cost demands. In fact, as energy for heating is not involved in RO systems, the magnitude of the performance ratio is much smaller than that of MD. The only limitation of this option is its lower salt rejection rate compared to MD: the salt rejection rate of RO is limited to 65%. Fig 11 demonstrates that there is an increasing operating pressure demand when the RO feed is constant, but a lower pressure is required when the feed rate increases. Salt separation becomes easier when the feed rate rises, as feed salinity is marginally influenced by the type of feed flow, e.g., constant or varying. Furthermore, Fig 11 shows the superior effect of using a varying makeup compared with using a fixed makeup, as a higher recovery rate is obtained at a lower cost and use of energy per capita. The enhanced recovery rate can be attributed to the diminishing fresh feed flow rate, and the improvements in specific energy and cost are related to the lower pumping energy demand incurred with respect to the fixed feed flow rate and the insignificant difference in the operating pressure.

**Fig 12:**
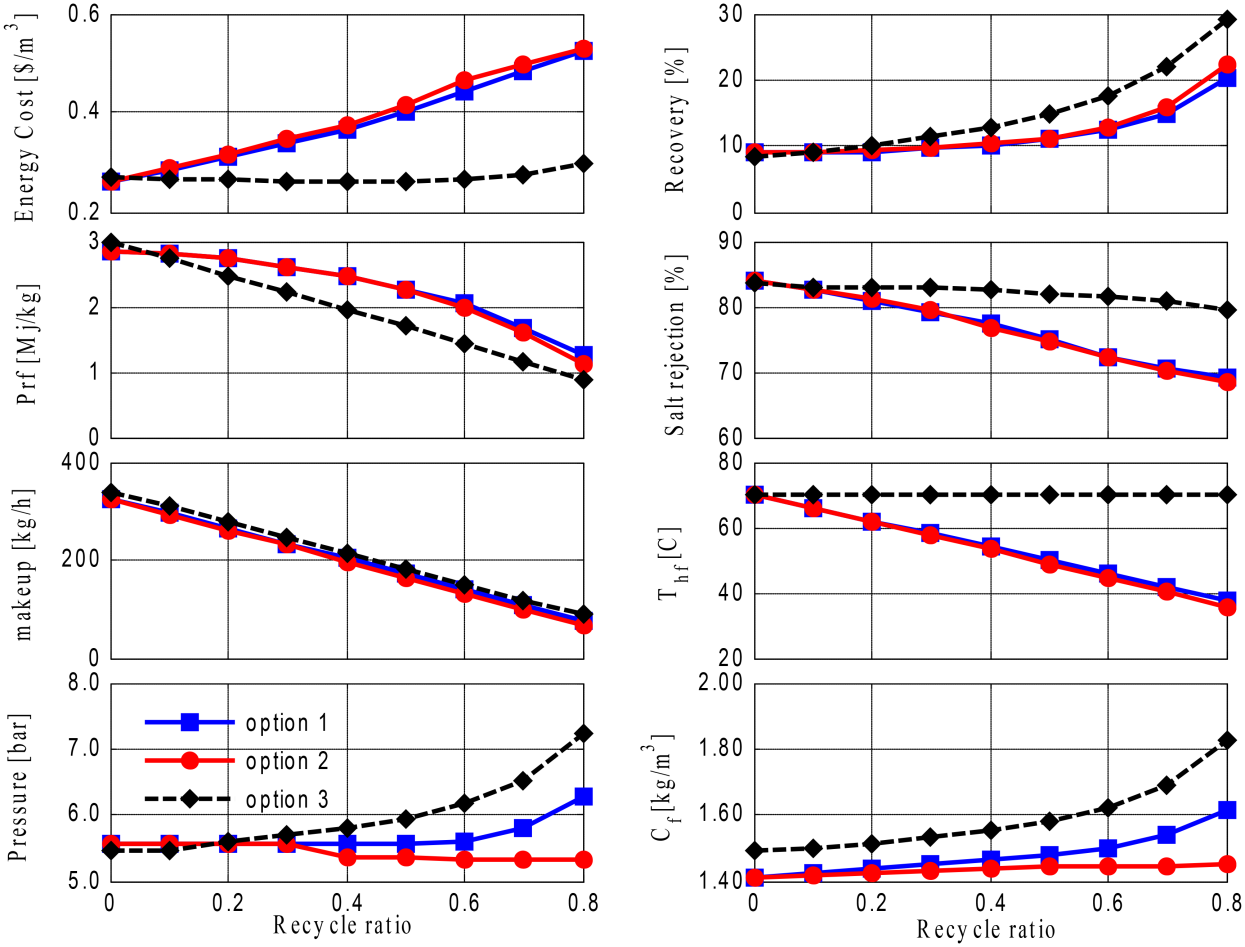
Performances of Options 3 to 5 with varying fresh feed.

We then investigated improving the performance of Option 2 by integrating RO and MD, thereby utilizing both the geothermal energy associated with the brackish water and the ability of the MD to produce high-quality water. Fig 12 compares Options 3, 4, and 5 where a varying makeup is used; (the makeup decreases with an increase in recycling). It is evident that Option 5, (in which brine is recycled around the RO unit) outperforms the other configurations, and a higher recovery ratio is obtained that reaches 30% at an 80% recycling ratio. The inferior recovery rates of Options 3 and 4 are caused by the recycling of cold brine, which decreases the main driving force of the MD process. Moreover, Option 5 requires a much lower specific operating cost than Options 3 and 4; its specific energy cost varies only marginally with higher brine recycling. Although the pumping energy employed is almost the same for the three cases, the ratio of the pumping cost to the predicted rate is higher and increases for Options 3 and 4, because they provide a decrease in production due to the loss of a driving force, as previously mentioned. The total energy consumption per capita (Pfr) decreases with the recycle ratio for all cases, but it decreases at a slower rate for Options 3 and 4. The heating energy, which is the dominant energy consumed in these cases, decreases proportionally with the makeup flow rate. However, because the water production of Options 3 and 4 increases at a slower rate than the water production of Option 5, the corresponding specific energy consumption decreases at a slower rate. It is of note that the RO operating pressure is slightly higher for Option 5, especially at a large recycling ratio, because the RO feed has higher salt concentration, as shown by the profile of C_f_. The salinity of the RO feed is lower in Options 3 and 4 than in Option 5, because the brine feedback in Options 3 and 4 mixes with a less saline stream (i.e., the makeup stream). It is of note that the higher-pressure demand of Option 5 does not affect the total energy consumption manifested by Pf, because the thermal energy demand prevails. Similarly, it does not have a considerable influence on the specific energy, because its contribution is diminished by the larger production rate.

**Fig 13:**
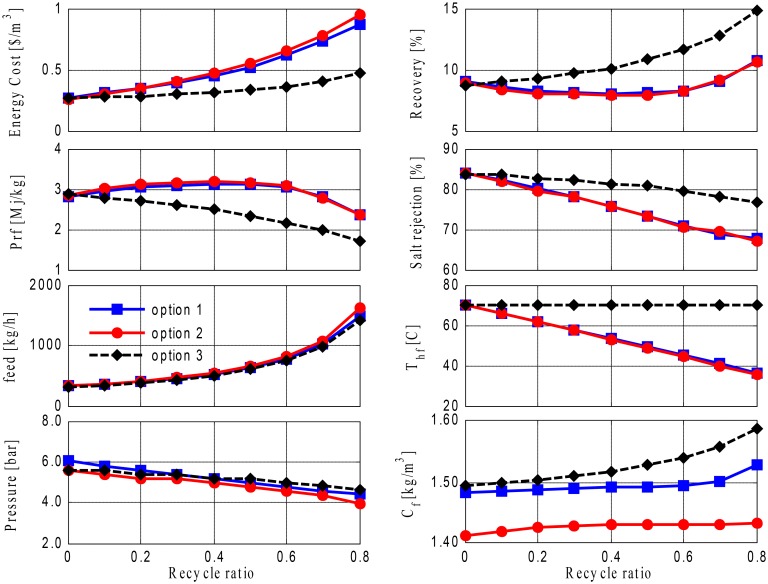
Performances of Options 3 to 5 with fixed fresh feed.

The performances of Options 3 to 5 were further compared using a fixed fresh feed (makeup) flow rate, but varying combined feed as in Fig 13. The superiority of Option 5 compared to the others can again be confirmed for the same reasons that have been previously discussed. However, the overall performance is not as good as that of the varying makeup case; for example, the maximum attainable recovery ratio for Option 5 is 15%. The reason for the lower recovery rate in this case is that the production rate is divided by the fixed makeup, whereas in the previous case, the production rate was divided by the decreasing makeup. In addition, the specific energy cost is higher in this case, because the pumping energy increases significantly due to the exponential increase in the total feed flow rate and not because of the lower production rate. The performance ratio is also higher for the same reasons. As shown in Fig 13, the required operating pressure for the RO process decreases slightly with the recycling ratio for all options; however, its contribution to the pumping energy demand, and consequently to the specific energy cost, is outweighed by the effect of the increasing feed flow rate. Overall, operating these design structures at varying makeups delivers more favorable performances in terms of higher recovery and lower operating costs and energy demands, compared to when the fixed makeup is used, and this result is considered to be reasonable, because the idea of recycling is to maximize production without exhausting resources.

**Fig 14:**
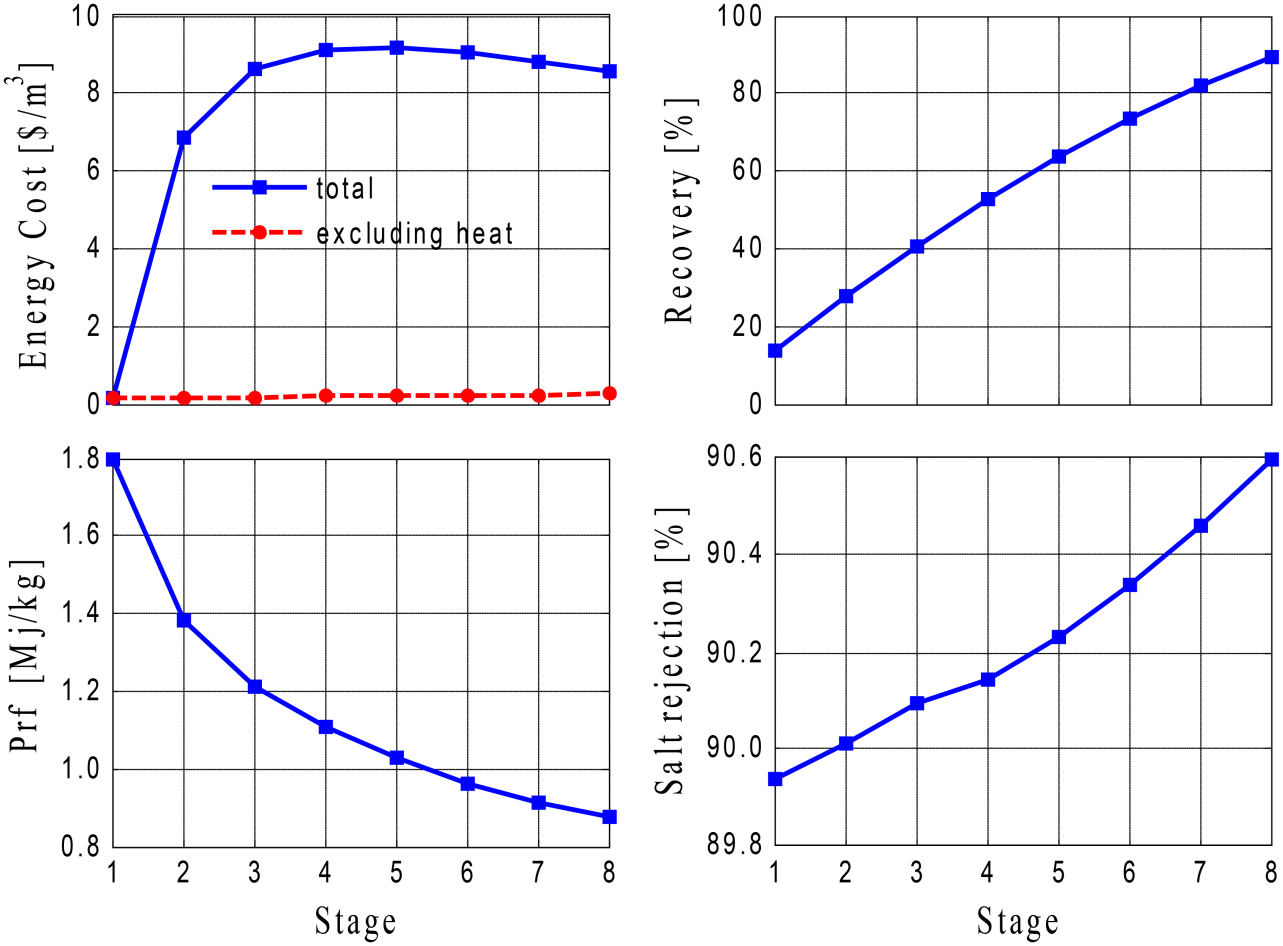
Performance of Option 6 using 8 stages.

An alternative method of maximizing water production is to employ a multi-stages concept, as shown in Fig 6. Connecting several stages in series is conceptually similar to recycling, because the rejected brine is reutilized. The simulation results for this design configuration are shown in Fig 14, in which the KPIs are plotted against the number of stages. The results indicate a considerable enhancement in the recovery rate, which approaches 90% when 8 stages are used. This value of the recovery ratio is superior to that provided by any of the other configurations. With an increase in the number of stages, the performance ratio and specific operating cost approach an asymptotic value, and when the largest number of stages is employed, the performance ratio obtained is comparable with that of Option 5 with a varying makeup. Moreover, these promising results are also obtained with an excellent salt rejection ratio of 90%. However, as external heating is used, these remarkable results are achieved at the expense of a much higher energy cost, which culminates at 9 $/m^3^, but if a free heat source (such as geothermal or waste energy) were used to preheat the feed, substantial savings would be obtained (as shown by the dashed line in Fig 14, which shows how specific energy is minimized when the heating demand is excluded).

**Fig 15:**
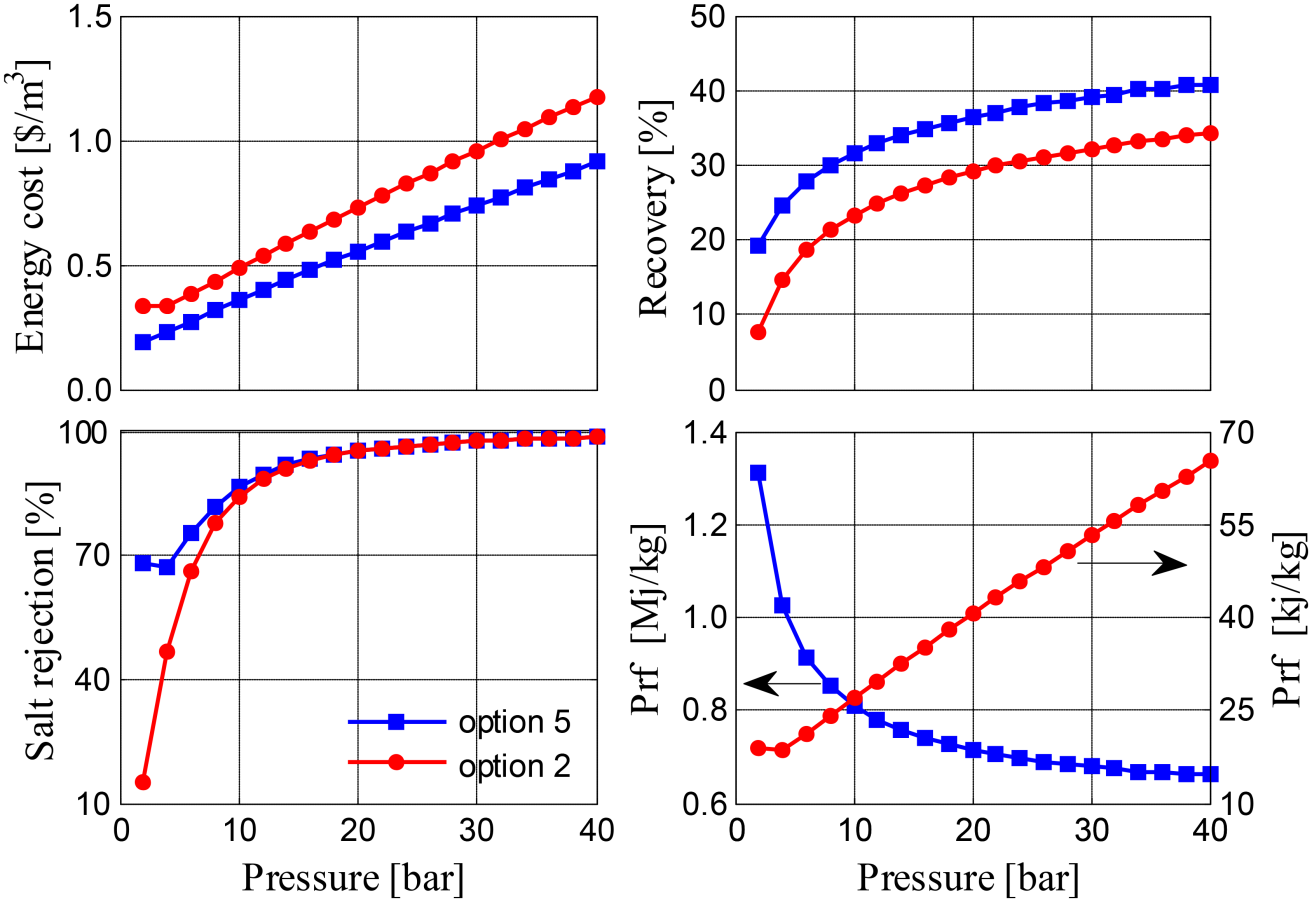
Performance of options 2 and 5 at various pressure values.

In the simulations above, the RO unit is operated at the minimum pressure necessary to achieve a good quality of distilled water, e.g., 0.5 kg/m^3^ salinity (500 ppm). However, we considered it would be interesting to examine the effect of increasing the operating pressure on performance. For this purpose, we compared the performance of configuration 5 with a varying makeup with that of configuration 2. We selected Option 5 with a varying makeup, because it delivered the best performance, and then compared it to Option 2, because the use of RO without MD is common practice in the local industry. The comparison is shown in Fig 15. Results show the benefits of using an integrated MD/RO system, as recovery rates are enhanced and specific energy costs are achieved. In fact, 40% recovery can be attained using Option 5 at an operating pressure of 40 bar, and this high recovery can be obtained at a reasonable specific cost of less than 1 $/m^3^. Both options produce high-quality water with 99% salt rejection. The performance ratio of Option 5 is improved substantially; however, the amount of pumping energy used increases because the total energy consumption is dominated initially by the thermal energy of the brackish water, and with a quick increase in the production rate relating to the increased pressure, the performance ratio declines readily. For Option 2, in which no thermal energy is involved, P_fr_ increases with pressure; however, it remains lower than in other options (note that it is quoted in kJ instead of MJ in Fig 15).

It is of interest that Choi et al. [28] recently evaluated the economic feasibility of RO, MD, and RO—MD hybrid systems for a fixed plant capacity of 50,000 m^3^/day. One of the main conclusions of this work is that the most important factor influencing the economics of MD and MD—RO systems is the cost of the thermal energy source. Therefore, for a low-cost energy source, the use of MD singly or RO—MD is attractive economically, especially when the recovery ratio is higher than that of single RO. Additionally, Choi et al. [28] estimated the total cost of water for RO, MD, and RO—MD systems at 0.75, 0.91, and 0.75 $/m^3^, respectively. These numbers are very close to those obtained in the present work, and we consider that the differences in the plant sizes used explains the small discrepancy.

## Conclusions

Geothermal brackish water is usually distilled using RO units after the treated feedstock has been cooled. However, there is much potential in using the thermal energy of geothermal water and employing MD, which provides the added advantages of producing high-purity water and being insensitive to feed salinity. In this study, different design configurations of MD/RO hybrid systems were investigated, which included differences in brine recycling and the cascading structure. The results substantiated the superiority of the integrated system compared to the conventional one; the recovery ratios ranged from 30% to 40% and the energy costs per m^3^ ranged from 0.4 to 0.9 $ when using variations in the RO operating pressure between 6 and 40 bar, respectively. It was found that brine recycling also improved the recovery rate and performance ratio only when brine was reused around the RO unit. When the rejected brine was fed back to the MD feed, the performance deteriorated because cold recycled brine quenched the MD feed and led to a reduced driving force inside the MD membrane. It was also revealed that using a multi-stage MD/RO interplay system connected in series enhanced the performance. In fact, a 90% recovery ratio and 0.9 MJ/kg performance ratio were obtained when 8 stages were employed. Although the production cost increased to 9 $/m^3^ because inter-stage heating was involved, if waste heat was used for inter-stage heating, the specific energy cost would be considerably reduced.

## Supporting information

S1 File (DOCX)

S2 File (XLSX)

S3 File (XLSX)
