# Supplementary material for: Enhancement of brackish water desalination using hybrid membrane distillation and reverse osmosis systems
Source: PLoS One. 2018 Oct 9;13(10):e0205012. doi: 10.1371/journal.pone.0205012 (PMC6177152; doi:10.1371/journal.pone.0205012)
Supplement: S1 File — (DOCX) [file pone.0205012.s001.docx]

**RO desalination unit model**

In the following, we present the basic equations governing the transport phenomena inherent to the RO process. These equations are provided in various studies [29,30]. For example, Marriott and Sorensen [30] developed the steady state equations describing the transport of salt and solvent in a typical RO membrane as

| $Q_{f}=Q_{p}+Q_{b},$ $Q_{f}C_{f}=Q_{p}C_{p}+Q_{b}C_{b}.$ | (S.1)  (S.2) |
| --- | --- |

The volumetric flux of solvent, *J*_w_, and the mass flux of solute, *J*_s_, are given by [31],

| $J_{w}=A\left( T \right)\left( \Delta P-\Delta\pi\right),$ | (S.3) |
| --- | --- |
| $J_{s}=B\left( T \right)\left( C_{m}-C_{P} \right).$ | (S.4) |

The following correlation for the pressure drop is used in Eq. (S.2) [31],

| $\Delta P=P_{f}-P_{b}-P_{drop}/2$, $\Delta\pi=b_{m}\left( C_{w}-C_{p} \right).$ | (S.5)  (S.6) |
| --- | --- |

The solvent flux, *J*_w_, at steady state is related to the concentration polarization as follows [32],

| $J_{w}=k_{s}ln\frac{C_{w}-C_{p}}{C_{b}-C_{p}}.$ | (S.7) |
| --- | --- |

The solute flux is used to determine the osmotic pressure across the membrane as follows,

| $J_{s}=J_{w}Cp$. | (S.8) |
| --- | --- |

Combining Eqs. (S.5, S.6) and eliminating *C*_m_ results in the following expression for the flux [33],

| $J_{w}=A(T)\left[ \Delta P-b_{\pi}\left( \frac{B\left( T \right)C_{b}\exp\left( \frac{J_{w}}{k_{s}} \right)}{J_{w}+B\left( T \right)\exp\left( \frac{J_{w}}{k_{s}} \right)} \right)exp(\frac{J_{w}}{k_{s}}) \right]$ | (S.9) |
| --- | --- |

and

| $C_{p}=\frac{B\left( T \right)C_{b}}{B\left( T \right)+J_{w}exp(\frac{J_{w}}{k_{s}})}$, | (S.10) |
| --- | --- |

$Q_{p}=J_{w}A.$ (S.11)

Once the nonlinear algebraic Eq. (S.7) is solved numerically, the concentration of the permeate, *C_p_*, can be calculated using Eq. (S.8). For the production of drinkable water, we constrain this concentration so that it is smaller than a desired specific value, *C_pd_*,

| $C_{p}\leq C_{pd}$. | (S.12) |
| --- | --- |

The dependence of the water permeability, *A*(*T*), and the salt permeability, *B*(*T*), of the membrane on temperature is given by the following [34],

| $A\left( T \right)=A_{0}\frac{\mu(T_{o})}{\mu(T)}$, | (S.13) |
| --- | --- |
| $B\left( T \right)=B_{0}\times\frac{T}{T_{o}}\times\frac{\mu(T_{o})}{\mu(T)}$. | (S.14) |

The viscosity, *μ*(*T*), can be calculated by the Guzman–Andrade equation [35],

| $\mu\left( T \right)=ae^{\frac{b}{T}},$ | (S.15) |
| --- | --- |

where the values of *a* and *b* can be calculated from the available correlations [36].

Perforated baffles are used in spiral-wound membrane modules because they increase mass transfer: the following equation can be used to determine the mass transfer coefficient, *k*_s_ [37],

| $Sh=0.065Re^{0.865}Sc^{0.25}$, | (S.16) |
| --- | --- |

where

(S.17)

The spiral-wound module hydraulic diameter depends on the specific surface area of the spacer, the void fraction, and the channel height. Table S.1 shows the membrane specifications [38].

The velocity in the feed channel that contains the baffle is given by

| $u=\frac{Q_{b}}{wh_{sp}\varepsilon}$, | **(S.18)** |
| --- | --- |

where *d*_h_, *h*_sp_, and *ε* are the baffle parameters. The kinematic viscosity, *ν*, for brackish water can be calculated through following correlation [39],

| $v=0.0032+3.0\times{10}^{-6}C_{b}+4.0\times{10}^{-9}C_{b}^{2}$. | (S.19) |
| --- | --- |

The value of diffusivity, *D*_AB_, is estimated to be 5.5 × 10^−6^ m^2^/h. Therefore, the osmotic coefficient, *b*_π_, is

| $b_{\pi}=\pi/C_{b}$. | **(S.20)** |
| --- | --- |

The osmotic pressure, *π*, is calculated using the following relationship,

| $\pi=1.12T\sum m_{i},$ | **(S.21)** |
| --- | --- |

where $\sum\bar{m}_{i}$is the sum of all molalities of dissolved ions (ppm). The energy needed to pressurize the feed stream is given by

| $E_{p}=Q_{f}P_{f}/\eta_{p}$. | **(S.22)** |
| --- | --- |

The water pressure on the concentrate side is

| $P_{b}=\Delta P-P_{drop},$ | (S.23) |
| --- | --- |

and the pressure drop, *P*_drop_, is given by the following correlation [31],

| $P_{drop}=\lambda\left( \frac{Q_{f}+Q_{b}}{2\times3600} \right)^{\alpha}$ | (S.24) |
| --- | --- |

In Eq. (S.24), λ = 9.5 × 10^8^ and *α *= 1.7.

The performance of the RO is assessed by selected key performance indices (KPI), such as the recovery rate (Rc), salt rejection (SR), performance ratio (P_fr_), and specific energy consumption. These KPIs are defined as follows,

| $Rc=Q_{p}/Q_{f}$, | (S.25) |
| --- | --- |
| $SR=1-Cp /C_{f},$ | (S.26) |
| $Pfr=\frac{E_{p}}{Q_{p}\rho}$, | (S.27) |
| $E_{cost}=\frac{E_{p}\times e_{c}}{Q_{p}}$. | (S.28) |

**Table S.1** Geometric specifications of RO membrane module [38].

| Parameter | Value |
| --- | --- |
| Hydraulic diameter of channel, *d*_h_ (mm) | 0.78045 |
| Height of spacer channel, *h*_sp_ (mm) | 0.593 |
| Void fraction of the spacer, ε (porosity) | 0.9 |
| Length of membrane, L (m) | 1 |
| Width of membrane, W (m) | 37 |
| Active area of membrane, A_e_ (m^2^) | 37 |
| Reference water permeability, A_0_ (m^3^/h·bar) | 19.43 × 10^−4^ |
| Reference solute permeability, B_0_ (m^3^/h) | 78.55 × 10^−5^ |

**DCMD desalination unit model**

The mass flux (*J*) of vapor transfer through pores is given by

$J=C_{m}\left( P_{1}-P_{2} \right)\left( \frac{\frac{kg}{m^{2}}}{s} \right)$. (S.29)

In Eq. (S.29), P_1_ and P_2_ are the partial pressures of water vapor estimated at the membrane surface temperatures T_mf_ and T_mp_, respectively. The partial pressure in Pa is estimated using the Antoine equation [39-41],

$P_{1}=\exp\left( 23.238-\frac{3841}{T_{hm}-45} \right)\left( 1-C_{s} \right)\left( 1-0.5C_{s}-10C_{s}^{2} \right),$ (S.30)

$P_{2}=\exp\left( 23.238-\frac{3841}{T_{cm}-45} \right),$ (S.31)

where *C_s_* is the water salinity in percentage. C_m_ is the MD coefficient calculated from three correlations depending on the type of mass transfer regime:

Knudson flow mechanism

$C_{m}^{k}=\frac{2\varepsilon r}{3\tau\delta}\left( \frac{8M_{w}}{\pi RT} \right)^{1/2}$, (S.32)

Molecular diffusion mechanism

$C_{m}^{D}=\frac{\varepsilon}{\tau\delta}\frac{PD}{P_{a}}\frac{M_{w}}{RT}$ , (S.33)

and Knudsen molecular diffusion transition mechanism

$C_{m}^{C}=\left[ {\frac{3}{2}\frac{\tau\delta}{\varepsilon r}\left( \frac{\pi RT}{8M_{w}} \right)}^{1/2}+\frac{\tau\delta}{\varepsilon}\frac{P_{a}}{PD}\frac{RT}{M_{w}} \right]^{-1}.$ (S.34)

These different regimes depend on the wall collision theory of water molecules, and each regime dominates at a specific range of values for the mean free path of a water molecule. The heat transfer process occurs in three steps:

1. Convection from the feed bulk to the vapor–liquid interface at the membrane surface,

$q_{f}=h_{f}\left( T_{h}-T_{mh} \right)$; (S.35)

1. Convection from the vapor–liquid interface at the membrane surface to the permeate side,

$q_{p}=h_{p}\left( T_{mc}-T_{c} \right);$ (S.36)

where *h*_f_ and *h*_p_ denote the heat transfer coefficients on the feed and cold-stream sides, respectively, and

1. Evaporation and conduction through the microporous membrane,

$q_{m}=JH_{v}+h_{m}(T_{mh}-T_{mc})$, (S.37)

where H_v_ is the water latent heat, which can be estimated using Eq. (S.38) [42]; and *h*_m_ is the conductive heat transfer coefficient, which is equal to *k*_m_/δ (where *k*_m_ and δ denote the membrane thermal conductivity and the membrane thickness, respectively),

$H_{v}\left( T \right)=1850.7+2.8273T-1.6\times{10}^{-3}T^{2}$. (S.38)

The total heat flux across the membrane is directly proportional to the bulk temperature gradient and can be expressed as follows,

$H=U\left( T_{h}-T_{c} \right).$ (S.39)

For countercurrent flow, the bulk temperatures are taken as $T_{h}={T_{h}}_{in}, T_{c}={T_{c}}_{out}$.

The overall heat transfer coefficient is given by

$U=\left[ \frac{1}{h_{f}}+\frac{1}{h_{m}+\frac{JH_{v}}{T_{mh}-T_{mc}}}+\frac{1}{h_{p}} \right]^{-1}$. (S.40)

Under steady-state operation, the heat transfer in the three individual parts of the system reaches equilibrium,

$q_{f}=q_{m}=q_{p}$ . (S.41)

Considering the macroscopic scale of the MD unit (Fig. S.1), the heat balance around the permeate side is given by [43],

**Fig. S.1.** **Typical DCMD unit.**

$UA_{s}\left( T_{h}-T_{c} \right)= Q_{c}\rho\mathrm{Cp}\left( T_{c_{\mathrm{out}}}-T_{c_{\mathrm{in}}} \right)$, (S.42)

where *Q_c_* and Cp denote the volume flow rate and the specific heat at constant pressure, respectively. Equation (S.42) is used to compute the permeate exit temperature, ${T_{c}}_{out}$. Similarly, assuming a constant density and heat capacity, the mass and heat balance around the feed side are given by

$UA_{s}\left( T_{h}-T_{c} \right)= Q_{h_{in}}\rho\mathrm{Cp}\left( T_{h_{\mathrm{out}}}-T_{\mathrm{ref}} \right)-Q_{h_{out}}\rho\mathrm{Cp}\left( T_{h_{\mathrm{in}}}-T_{\mathrm{ref}} \right),$ (S.43)

${Q_{h}}_{in}-{Q_{h}}_{out}=Q_{w}$, (S.44)

where Eq. (S.43) can be used for computing ${T_{h}}_{out}$. It should be noted that Eqs. (S.42) and (S.43) are based on an ideal case in which heat losses are negligible. An additional term can be added to account for heat losses as a percentage of the total heat transfer to make the calculated ${T_{c}}_{out} \mathrm{and} {T_{h}}_{out}$ match experimental values. Throughout the manuscript, ${Q_{h}}_{in}$ is also defined as the hot feed flow, *Q_hf_*; ${Q_{h}}_{out}$ is defined as the brine flow, *Q_r_*; ${T_{h}}_{in}$ is defined as the hot feed temperature, T_hf_; and ${T_{h}}_{out}$ is defined as the brine temperature, T_b_. The definitions of the various variables, the numerical values of the physical and design parameters in Eqs. (S.29–S.44), and additional supporting correlations are provided in [44,45].

The KPI for the MD process can also be defined as follows,

| $Rc=\frac{Q_{w}}{Q_{hf}},$ | (S.45) |
| --- | --- |
| $SR=1$, | (S.46) |
| $Pfr=\frac{E_{p}+H_{in}}{Q_{w}\rho}$, | (S.47) |
| $E_{cost}=\frac{E_{p}\times e_{c}}{Q_{w}}.$ | (S.48) |

The pumping energy in the MD unit is taken as follows,

$E_{p}=\left( {Q_{h}}_{in}+{Q_{c}} \right)\times1.5\times{10}^{5}/\eta_{p}$. (S.49)

Note that in Eq. (S.49), the pumping pressure is taken as the MD operating pressure of atmospheric pressure plus a pressure drop of 0.4 atmospheric pressure across the MD membrane. Furthermore, in Eq. (S.48), the cost of the heating utility is not included because it is inherent within the geothermal water.
